# Highly efficient *Agrobacterium rhizogenes*-mediated hairy root transformation in citrus seeds and its application in gene functional analysis

**DOI:** 10.3389/fpls.2023.1293374

**Published:** 2023-10-31

**Authors:** Min Wang, Yang-Yang Qin, Nan-Nan Wei, Huan-Ying Xue, Wen-Shan Dai

**Affiliations:** China-USA Citrus Huanglongbing Joint Laboratory, National Navel Orange Engineering Research Center, College of Life Sciences, Gannan Normal University, Ganzhou, Jiangxi, China

**Keywords:** citrus, genetic transformation, *Agrobacterium rhizogenes*, subcellular localization, gene editing

## Abstract

Highly efficient genetic transformation technology is beneficial for plant gene functional research and molecular improvement breeding. However, the most commonly used *Agrobacterium tumefaciens*-mediated genetic transformation technology is time-consuming and recalcitrant for some woody plants such as citrus, hampering the high-throughput functional analysis of citrus genes. Thus, we dedicated to develop a rapid, simple, and highly efficient hairy root transformation system induced by *Agrobacterium rhizogenes* to analyze citrus gene function. In this report, a rapid, universal, and highly efficient hairy root transformation system in citrus seeds was described. Only 15 days were required for the entire workflow and the system was applicable for various citrus genotypes, with a maximum transformation frequency of 96.1%. After optimization, the transformation frequency of *Citrus sinensis*, which shows the lowest transformation frequency of 52.3% among four citrus genotypes initially, was increased to 71.4% successfully. To test the applicability of the hairy roots transformation system for gene functional analysis of citrus genes, we evaluated the subcellular localization, gene overexpression and gene editing in transformed hairy roots. Compared with the traditional transient transformation system performed in tobacco leaves, the transgenic citrus hairy roots displayed a more clear and specific subcellular fluorescence localization. Transcript levels of genes were significantly increased in overexpressing transgenic citrus hairy roots as compared with wild-type (WT). Additionally, hairy root transformation system in citrus seeds was successful in obtaining transformants with knocked out targets, indicating that the *Agrobacterium rhizogenes*-mediated transformation enables the CRISPR/Cas9-mediated gene editing. In summary, we established a highly efficient genetic transformation technology with non-tissue-culture in citrus that can be used for functional analysis such as protein subcellular localization, gene overexpression and gene editing. Since the material used for genetic transformation are roots protruding out of citrus seeds, the process of planting seedlings prior to transformation of conventional tissue culture or non-tissue-culture was eliminated, and the experimental time was greatly reduced. We anticipate that this genetic transformation technology will be a valuable tool for routine research of citrus genes in the future.

## Introduction

Citrus is one of the most valuable fruits worldwide and has been cultivated in China for more than 4000 years (Wu et al., 2014; Wu et al., 2018). At present, the citrus industry has become a mainstay for rural economy of the main production areas in the south of China, which has contributed positively to promoting the income of farmers, expanding the employment of urban and rural residents, and improving the ecological environment (Dahro et al., 2023). Since most citrus varieties are polyembryonic and asexual embryos develop superiorly to sexual embryos, the efficiency of citrus breeding through sexual crosses is relatively low (Zhang et al., 2018). The characteristics of both prolonged juvenile period and abortion of female/male organs severely limit the research on gene functional and biological breeding of citrus plants (Wu et al., 2021). This puts forward more demands on the technical means of citrus-related research, especially the urgent need for the establishment of an efficient and rapid genetic transformation system in citrus.

In recent years, *Agrobacterium tumefaciens* (*A. tumefaciens*)-mediated genetic transformation system in citrus epicotyl has been successfully established and applied (Wang et al., 2019; Ramasamy et al., 2023). However, the research progress is sluggish due to long transformation period and unstable efficiency, especially for the functional research of root-related genes and the efficiency evaluation of gene editing system, and possesses the disadvantages of time-consuming and high-risky, which limits its application. While, the more recently studied *Agrobacterium rhizogenes* (*A. rhizogenes*)-mediated hairy root transformation system is a rapid and efficient research technology that compensates for the long and unstable period of genetic transformation mediated by *A. tumefaciens*, or the low transformation efficiency of some *A. tumefaciens*-unaffiliated plants (Kereszt et al., 2007; Irigoyen et al., 2020; Cheng et al., 2021). The establishment of hairy root transformation system is achieved by the Gram-negative bacterium, *A. rhizogenes*, which enables researchers to infect most dicotyledonous and a few monocotyledonous species, as well as individual gymnosperms (Castellanos-Arévalo et al., 2020). A transfer DNA (T-DNA) on Ri plasmid from *A. rhizogenes*, which encodes multiple *root locus* (*rol*) genes presumed to promote cell dedifferentiation into roots, is introduced and integrated into the plant genome during infection, thus inducing the formation of infected plant to hairy roots with multiple branches at the wound (Qu and Christ, 2007). Therefore, *A. rhizogenes-*mediated transformation technology has great potential for application in studying plant gene function and biological characteristics, due to the fast growth rate, non-requirement of exogenous hormone supplementation, high genetic stability and excellent ability to synthesize secondary metabolites of transgenic hairy roots.

Currently, both tissue culture or non-tissue-culture systems of hairy root transformation have been exploited in a variety of plants (Estrada-Navarrete et al., 2007; Fan et al., 2020), but researches on transgenic hairy roots in citrus have lagged relatively behind. Previous studies have mostly used mature branches or seedlings as the explants for hairy root induction (Ma et al., 2022), which possesses the high differentiation and the low induction efficiency, as well as the time-consuming for pre-grown of seedlings, resulting in slow experimental progress. In this study, we describe a rapid, universal, and highly efficient hairy root transformation system mediated by *A. rhizogenes* in citrus seeds for the first time. The entire workflow requires only 15 days and the system was applicable for various citrus genotypes. Transgenic hairy roots can be used for subcellular localization, gene overexpression analysis, and CRISPR/Cas9-mediated gene editing researches. We anticipate that this genetic transformation technology using citrus seeds as explants will provide a powerful tool for functional studies of citrus genes.

## Materials and methods

### Plant materials and growth condition

Seeds of four citrus genotypes, *Citrus sinensis* (*C*. *sinensis*), *Poncirus trifoliata* (*P*. *trifoliata*), *Citrus limon* (*C*. *limon*), and *Citrus grandis* (*C*. *grandis*), were used in this study. Three hundred seeds of each genotype were cleaned with distilled water, followed by soaking in 1 M NaOH solution for 15 min to remove pectin, and then rinsed 1-2 times with sterilized water. The cleaned seeds were spread on sterilized and moistened gauze and placed in an incubator at 30°C approximately 7 days for germination. Young radicles of citrus seeds sprouting to 1-2 cm long were selected for transformation.

### *Agrobacterium* strains and vector constructions

The *A. rhizogenes* (K599) competent cells (CAT#: CC410) and *A. tumefaciens* (GV3101) competent cells (CAT#: CC405) were obtained from beijing coolaber science & technology Co., Ltd. *A. rhizogenes* K599 was used to induce hairy roots. *A. tumefaciens* GV3101 was used in infltration assays with *Nicotiana benthamiana* (*N*. *benthamiana*). All *Agrobacterium* strains were stored in 20% glycerol and preserved at -80°C in the refrigerator.

For assessment of the efficiency of hairy root transformation system in citrus seeds, a binary plasmid pCAMBIA1380 possessing a green fluorescent protein (GFP) protein was used. For subcellular localization experiments, three citrus endogenous proteins, CsWRKY7, CitSWEET6, and CitF3’H, each fused with GFP protein were employed as the nucleus marker, plasma membrane marker and endoplasmic reticulum (ER) marker, respectively (Liu et al., 2022; Dai et al., 2023; Fang et al., 2023). For overexpression analysis, the coding region of *CsWRKY17* (1026 bp) was cloned into the pCAMBIA1300-GFP binary vector driven by the cauliflower mosaic virus 35S promoter (CaMV 35S). For gene editing experiments, a phloem protein gene from *C. sinensis*, *CsPP2-1* (Cs_ont_2g009390), was selected as a reference gene to construct the CRISPR vector. The gRNA-targeted loci were designed based on the genomic sequence of *CsPP2-1* using the CRISPRP v2.0 (http://crispr.hzau.edu.cn/cgi-bin/CRISPR2/CRISPR). To ensure editing efficiency, two gRNA-target sites of 20 bp were designed in the first and second exons of the *CsPP2-1*, respectively. Double-stranded gRNA1/gRNA2 fragments formed by annealing were primordially ligated into AtU6-26-sgRNA-SK vector (predigested by *Bsa* I), respectively (**Table S1**). Then, the AtU6-26-gRNA1-sgRNA and AtU6-26-gRNA2-sgRNA cassette were cut off by *Spe* I and *Nhe* I, and co-ligated into pCAMBIA1300-p*YAO*-Cas9 vector (predigested by *Spe* I) harboring Cas9 and GFP proteins successively. This design will increase the possibility of affecting protein function and the likelihood that at least one site would be edited.

### *A rhizogenes-*mediated hairy root transformation in citrus seeds

The *A. rhizogenes* used for transformation was cultured in 50 mL fresh LB liquid medium (Tryptone 10 g/L, Yeast extract 5 g/L, NaCl 5 g/L) containing the corresponding antibiotics at a ratio of 1:100 and incubated for 12 h incubated at 28°C with shaking at 200 rpm until a suitable optical density at 600 nm (OD_600 =_ 0.6-0.8) was reached. Then, the K599 cells were centrifuged at 4000 rpm for 10 min, followed by resuspension in 2-(N-morpholino) ethanesulfonic acid (MES) buffer (10 mM MgCl_2_, 10 mM MES, and 100 μM acetosyringone, pH 5.6), and ensured that the OD_600_ values were consistent with those before centrifugation.

The germinated radicles that were 1-2 cm long were selected and punctured with needles sterilized with 70% ethanol to increase the wounding surface for *A. rhizogenes* inoculation. Young roots were punctured at about 5-8 wound points per centimeter all around to a depth of about 0.1-0.3 cm. The seeds with punctured roots were completely immersed in *Agrobacterium* mixtures and permeated under vacuum infiltration for 10 min at 0.08-0.09 MPa. The germinated seeds were laid flat on filter paper moistened with sterile water and incubated in the dark at 25°C for 3 d. Afterwards, the citrus seeds co-incubated with *A*. rhizogenes were placed in a tray filled with vermiculite at a 25°C incubator with 90% relative humidity and a 16: 8 h, light: dark conditions to produce hair roots. Hairy root development began after approximately 10-15 days after agroinfiltration, and abundant hairy roots could be observed 30 days after inoculation. Potential positive transgenic roots were detected by a portable fluorescent protein excitation light source (Luyor-3415RG, Shanghai, China). The hairy root transformation efficiency (positive rates) was calculated using the following formula: [(Number of GFP-containing roots)/(Total number of roots)] × 100.

### *A*. *tumefaciens* infiltration assays with *N. benthamiana*

The *A. tumefaciens* GV3101 carrying each fusion construct were cultured in LB medium with corresponding antibiotic for 12-16 h at 28°C. The strains were pelleted by centrifugation for 15 min at 4000 rpm and resuspended to a cell density of OD_600 =_ 0.8 in a MES buffer (10 mM MgCl_2_, 150 μM acetosyringone, and 10 mM MES, pH 5.6). Tobacco (*N. benthamiana*) leaves were injected with GV3101, as has been described previously (Zhang et al., 2023). The infiltrated plants were grown for an additional 3 days prior to fluorescence signal detection.

### Confocal microscopy observation

Transgenic citrus hairy roots and *N. benthamiana* leaves were prepared for fuorescence observations under a confocal laser scanning microscope (Leica TCS SP8, Germany) with the 488 nm excitation wave-lengths for GFP fuorescence.

### Positive identification of overexpressing transgenic hairy roots

Genomic DNA was extracted from positive overexpressing citrus hairy roots using the cetyltrimethylammonium bromide (CTAB) method. The specific primers used for positive identification were designed using Primer Premier 5.0 software (**Table S1**). Cirus DNA that could be amplified with target fragments by three pairs of specific primers (NPTII, GFP and gene-specific primers) were considered to be from positive overexpressing hairy roots, which were retained for qRT-PCR to detect the transcript abundance.

### RNA extraction and quantitative real-time PCR analysis

Total RNA was extracted from hairy roots in accordance with the manufacturer’s instructions of the TaKaRa miniBEST universal RNA extraction kit (Takara, Japan). The quality and integrity of the total RNA were examined with agarose gel electrophoresis and the NanoDrop 2000. The cDNA was synthesized using PrimeScript^™^ RT reagent Kit with gDNA Eraser (Takara, Japan). The specific primers used for expression level detection were designed using Primer Premier 5.0 software (**Table S1**). Real-time qRT-PCR analysis was done using 2×TSINGKE^®^ Master qPCR Mix (TSINGKE, China) on a QuantStudio 5 Applied BioSystem (ThermoFisher Scientific, USA). Citrus *Actin* were used as internal reference genes for normalization of the relative expression level of the target genes. Each reaction was repeated in three biological replications, and the 2^-△△Ct^ method was applied to calculate the relative expression levels.

### Mutagenesis analysis with gene editing

Genomic DNA of positive gene-editing hairy roots was extracted as a PCR template. The target site and its nearby sequence were amplifed by PCR using the *CsPP2-1* specific primers: *CsPP2-1* CRISPR F and *CsPP2-1* CRISPR R (**Table S1**). 3 µL of amplification product was taken for agarose gel electrophoresis, and the product with the correct amplification size was ligated to the pTOPO-Blunt vector (Aidlab, China) for sanger sequencing to evaluate the mutation efficiency at the gRNA-target site. The sequence chromatograms were analyzed with SnapGene software.

### Statistical analysis

Each experiment was conducted at least three times independently. Statistical significance was determined using Tukey’s test at p < 0.05.

## Results

### A rapid and highly efficient strategy for *A. rhizogenes*−mediated hairy root transformation system in citrus seeds

**Figure 1:**
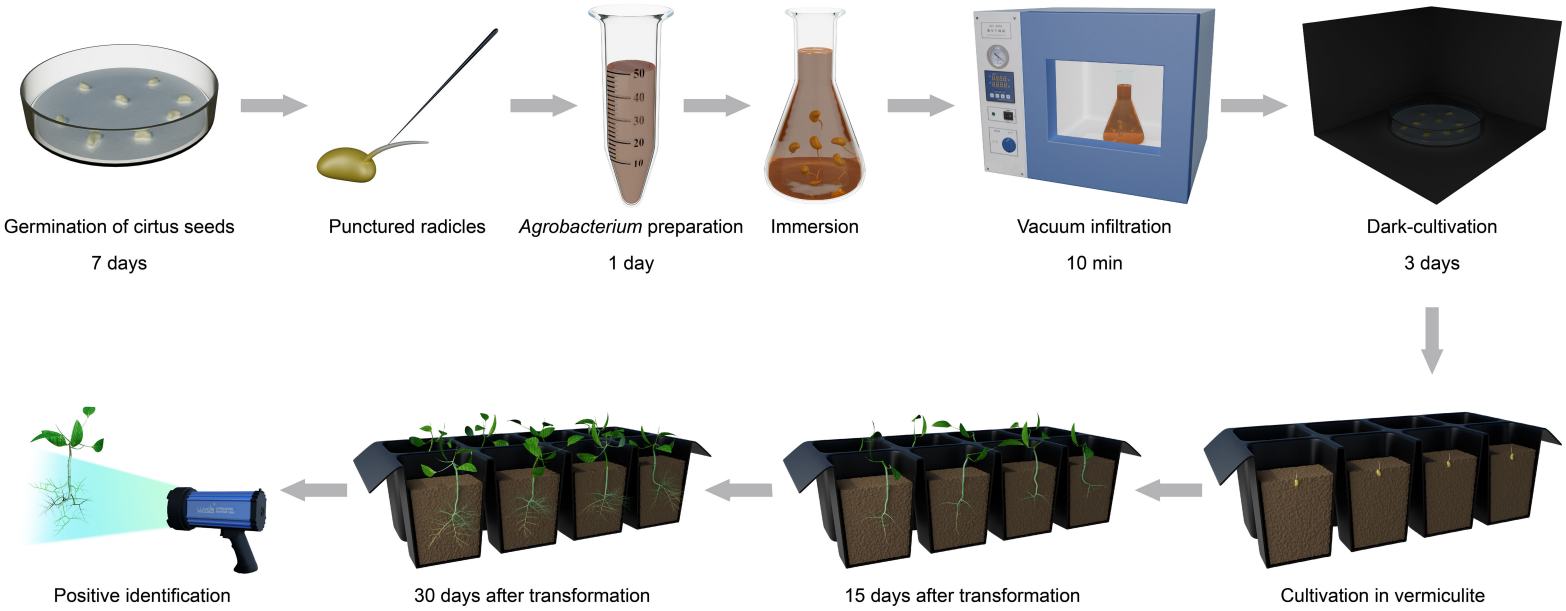
Workflow of the hairy root transformation for citrus by using seeds as explants.

Our goal was to establish a rapid, simple and highly efficient *A. rhizogenes*-mediated hairy root transformation system in citrus. Schematic presentation of our protocol is on the **Figure 1**.

**Figure 2:**
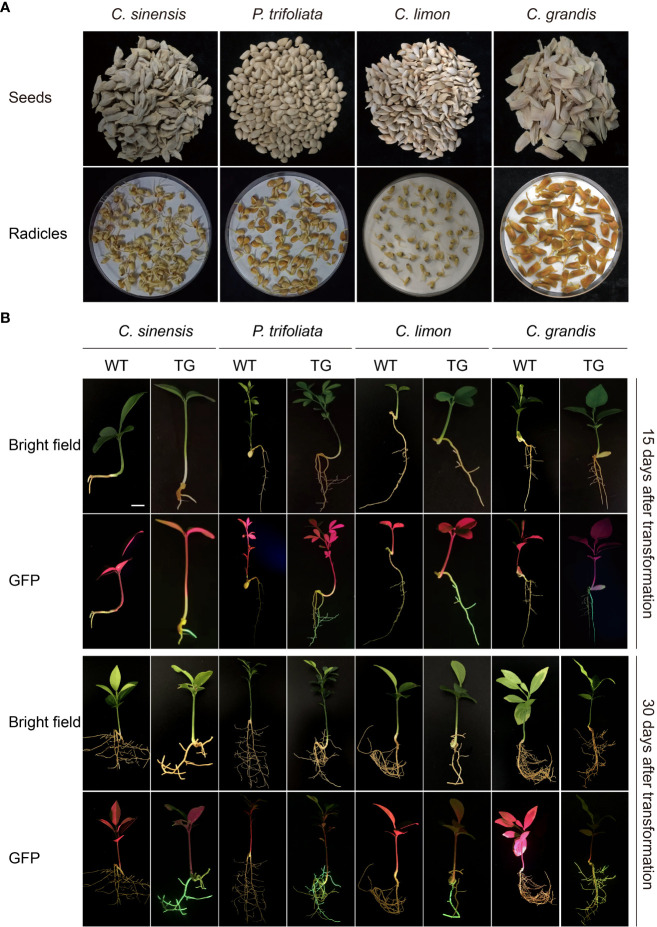
*A rhizogenes*-mediated hairy root transformation in citrus seeds. **(A)** Schematic diagram of the germination process of seeds from four citrus genotypes, namely *C sinensis*, *P*. *trifoliata*, *C limon* and *C grandis*. **(B)** Representative images of WT and transgenic hairy roots (TG) at 15 or 30 days post transformation. The scale bar indicates 1 cm.

Seeds of four citrus genotypes, *C*. *sinensis*, *P*. *trifoliata*, *C*. *limon*, and *C*. *grandis*, were selected for assessment of the efficiency of genetic transformation of citrus seeds mediated by *A. rhizogenes* K599 harboring a binary T-DNA vector with a GFP marker to induce hairy root growth (**Figure 2A**). The seedlings began to grow aboveground about 2 weeks after transformation, the statistics of germination rates showed that there existed differences in seed germination rates among citrus genotypes, which may be related to the preservation conditions and quality of respective seeds (**Table 1**). Usually, hairy roots emerged at 15 days post-inoculation, abundant hairy roots could be obtained 30 days after transformation for subsequent experiments (**Figure 2B**). Due to the presence of GFP protein, a portable fluorescent protein excitation light source (Luyor-3415RG, Shanghai, China) can be used for observation and detection of positive materials based on the emission of green fluorescence of the hairy roots. Non-transgenic WT seeds also germinate at similar phases, but can be easily distinguishable due to the lack of GFP signal. After 30 days of transformation, the positive rates of hair roots were analyzed, with *C. limon* hair roots possessing the highest positive rate of 96.1%. *C*. *grandis* and *P*. *trifoliata* owned second grade positive rate of 90.8% and 89.5%, respectively. *C*. *sinensis* had a positive rate of 52.3%, indicating that all four citrus genotypes successfully and efficiently obtained positive roots by using *A. rhizogenes*−mediated hairy root transformation system (**Figure 2B**, **Table 1**).

**Table 1:** Germination rate and root transformation positive rate of seeds from four citrus genotypes.

Genotypes	Germination rate (%)	Positive rate (%)
*C*. *sinensis*	61.6	52.3
*P*. *trifoliata*	43.0	73.8
*C*. *limon*	30.7	91.6
*C*. *grandis*	71.8	90.5

### Optimization of hairy root transformation system in *C. sinensis* seeds

**Figure 3:**
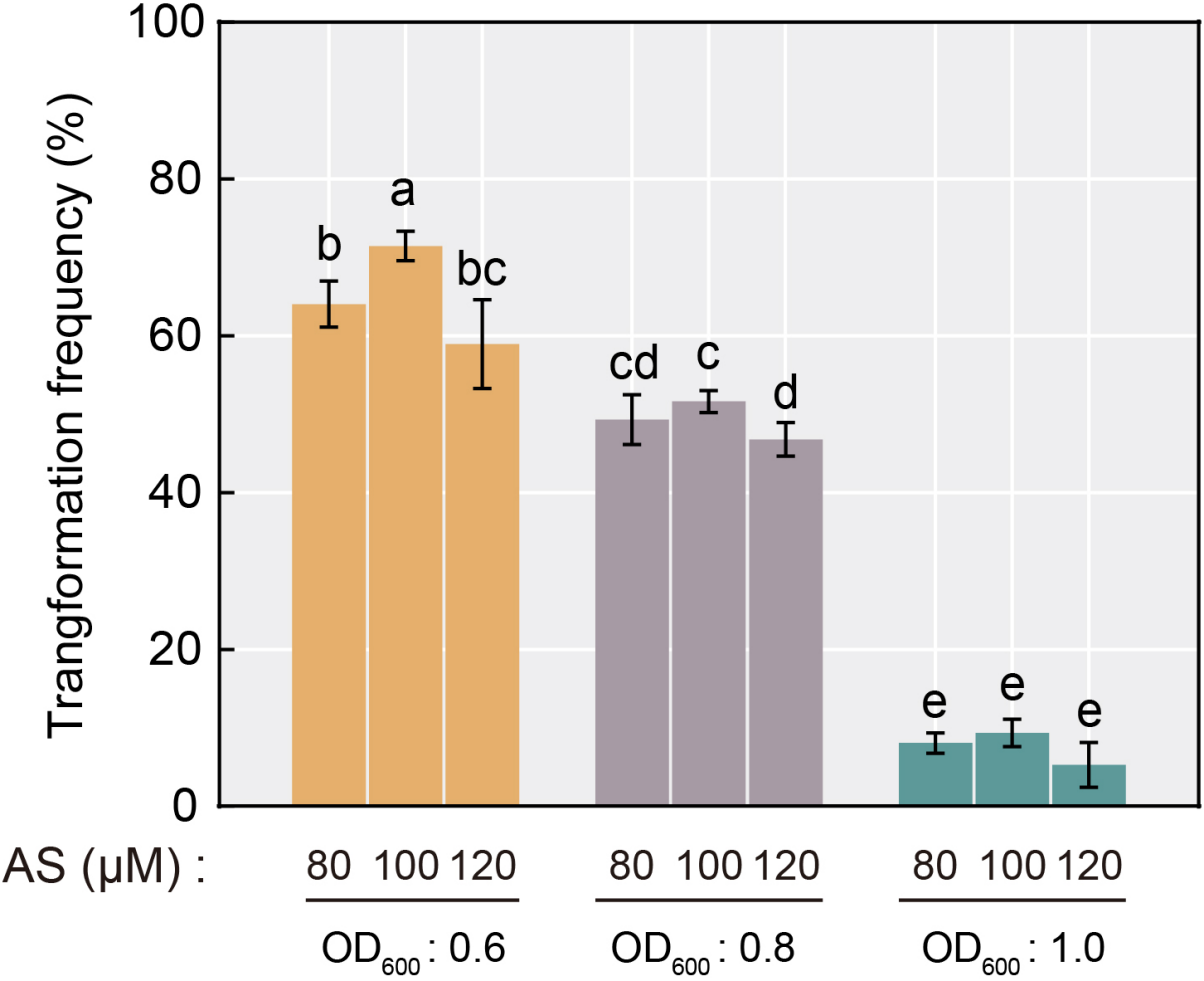
Transformation frequency of *A*. *rhizogenes*-mediated hairy root transformation in *C*. *sinensis* seeds with different AS concentration and *Agrobacterium* concentration selections. Different letters on top of the bars indicate significant differences among groups based on a Tukey’s test (*p*<0.05).

As one of the largest and most economically valuable species in citrus, *C*. *sinensis* possesses a relatively low genetic transformation efficiency compared to other species (Khan et al., 2012), and similar rates were obtained with the hairy root transformation system. To optimize the hairy root transformation efficiency in *C*. *sinensis* seeds, we explored the effect on transformation efficiency during the process by setting up a gradient of *A. rhizogenes* concentrations (based on OD_600_ value) and AS concentrations. The results showed that the frequency of transformation of *C*. *sinensis* mediated by *A. rhizogenes* exhibited a stepwise decrease with increasing *Agrobacterium* concentration. The positive rate ranged from merely 5.3% to 9.3% when the OD_600_ value of *Agrobacterium* reached 1.0. Under the same concentration of *Agrobacterium*, a tendency of increasing and then decreasing effect of the AS concentration utilized in the transformation process on the positive rate was observed. When the AS concentration reached 100 μM, the transformation efficiency remained maximum compared to AS concentrations of 80 μM and 120 μM, reaching up to 71.4% (**Figure 3**). Therefore, the *Agrobacterium* concentration of OD_600 =_ 0.6 and the 100 μM AS concentration were optimal for *A. rhizogenes*-mediated hairy root transformation in *C*. *sinensis*, and used in subsequent experiments.

### Application of hairy root transformation system in citrus seeds for protein subcellular localization

**Figure 4:**
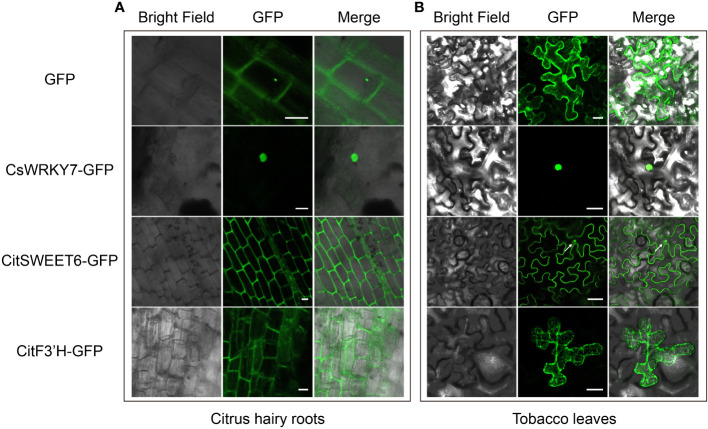
Protein subcellular localization with *C sinensis* hairy roots and tobacco leaves. GFP expression and subcellular localization with *C sinensis* hairy roots [left, **(A)**] and tobacco leaves [right, **(B)**] severally. Three citrus endogenous proteins, CsWRKY7, CitSWEET6, and CitF3’H, each fused with GFP protein were employed as the nucleus marker, plasma membrane marker and ER marker, respectively. Fluorescence was detected by a confocal laser scanning microscope. The scale bars of *C sinensis* hairy roots and tobacco leaves represent 20 µm or 50 µm, respectively.

Subcellular localization of plant protein is important for charactering gene function, and is usually conducted in *N. benthamiana* leaves by *A*. *tumefaciens*-mediated transient transformation because of the simplicity of this system (Luan et al., 2021). However, some organisms exhibit species-specific protein subcellular localization. Therefore, it is more rigorous and reliable to analyze subcellular localization of citrus protein in citrus rather than in other species. To verify whether the hairy root transformation system in citrus seeds could be used for analysis of protein subcellular localization, we evaluated the subcellular localization of citrus-endogenous proteins in nucleus, plasma membrane and ER from citrus hairy root cells. An *CsWRKY7* gene encoding a protein localized to the nucleus was fused with GFP and transformed into citrus hairy roots and tobacco leaves (Dai et al., 2023). As shown in **Figures 4A, B**, CsWRKY7 exhibited nuclear localization in both citrus hairy root and tobacco leave cells. Compared to tobacco, the cells of citrus hairy root could be observed more clearly. CitSWEET6 from *Citrus reticulata Blanco* localized to cell membrane in citrus hairy roots, but to the membrane and nucleus in tobacco leaves (The white arrow on the **Figure 4B** shows the nuclear localization of CitSWEET6) (Fang et al., 2023). A CitF3’H protein fused with a GFP protein showed ER localization in both citrus hairy root and tobacco leave cells, and the localization observed in citrus hairy root was more specific (**Figure 4**) (Liu et al., 2022). Therefore, the citrus seed hairy root transformation system enables the evaluation of citrus protein subcellular localization with more accurate results compared to the transformation system performed in conventional tobacco leaves.

### Genetic overexpression transformation with hairy root transformation system in citrus seeds

**Figure 5:**
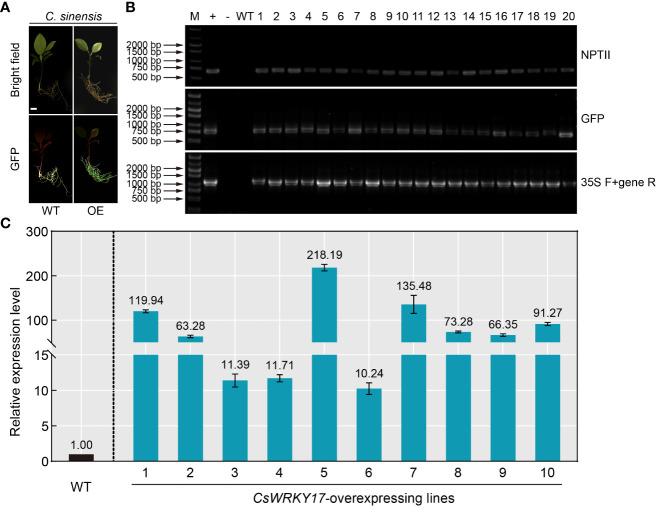
Positive identification and expression analysis of *A rhizogenes*-mediated overexpressing hairy roots. **(A)** Green fluorescence signal visualized in transgenic overexpressing roots (OE) of *C sinensis*. **(B)** Genomic PCR identification of the regenerated hairy roots using *NPTII*-specific primers (upper panel), *GFP*-specific primers (middle panel) and CaMV *35S*/*CsWRKY17* primers (lower panel). +: plasmid (used as a positive control); -: ddH_2_O; WT: wild-type, 1-20: transgenic lines of hairy roots. **(C)** Expression levels of *CsWRKY17* in WT and ten randomly selected transgenic hairy root lines. The scale bar indicates 1 cm.

Overexpression technology of foreign gene is a necessary and effective strategy for studying the gene function of plants (Zou et al., 2017; Song et al., 2022; Zhang et al., 2022). However, the commonly used *A*. *tumefaciens*-mediated transformation in citrus epicotyl usually takes several months to obtain transgenic seedlings. Given the high efficiency of *A. rhizogenes*-mediated hairy root transformation system in citrus seeds, the feasibility of *A. rhizogenes*-mediated overexpression was performed. An endogenous gene (*CsWRKY17*) was selected for overexpression transformation, and *C*. *sinensis* seeds were infiltrated with K599 harboring the *CsWRKY17*-pCAMBIA1300-GFP vector. After 30 days of transformation, PCR amplification was performed to verify the hairy roots emitting green fluorescence (**Figure 5A**). The DNA of all selected hairy roots contained sequences encoding NPTII protein and GFP protein, and the gene-specific primers were able to amplify the correct fragments (**Figure 5B**). No fragments were amplified by PCR from the WT roots, indicating that *CsWRKY17*-overexpressing vector was successfully transformed into hairy roots of *C*. *sinensis*. The qRT-PCR analysis revealed that the transcript abundance of *CsWRKY17* in ten randomly selected positive transgenic roots in comparison to WT, with the highest levels of 218.19-fold and the lowest of 10.24-fold (**Figure 5C**). Taken together, these results indicated that the *A*. *Rhizogenes*-mediated transformation system in citrus seeds could successfully introduce exogenous genes into citrus hairy roots and overexpress them.

### CRISPR/Cas9 gene-editing efficiency analysis with hairy root transformation system in citrus seeds

**Figure 6:**
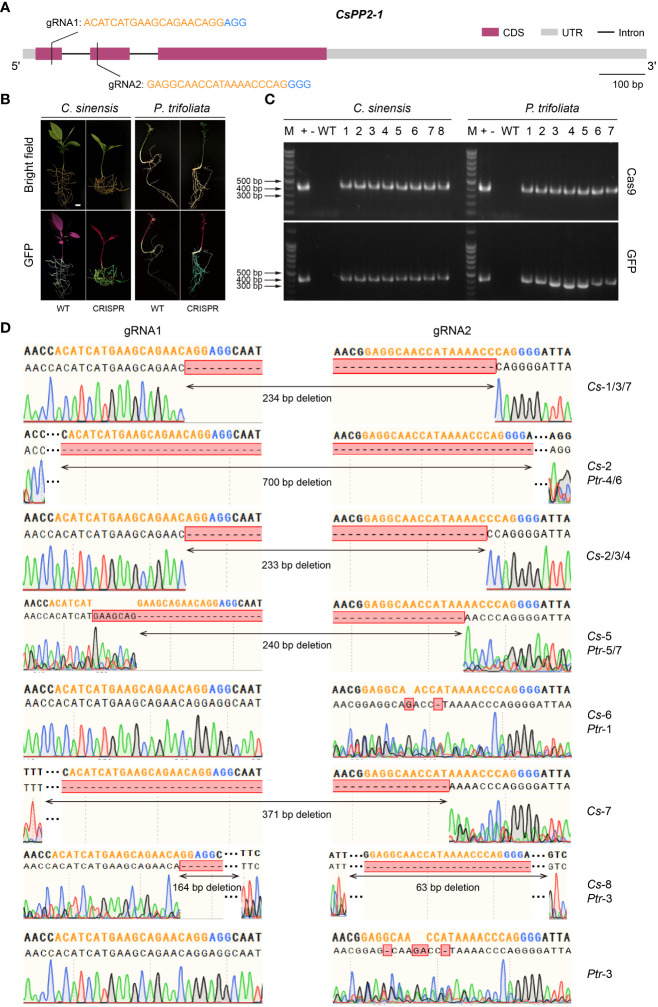
*A rhizogenes*-mediated gene editing in citrus seeds. **(A)** Gene structure and selected gRNA targets of *CsPP2-1*. Orange texts indicate gRNAs and blue texts indicate PAMs. **(B)** Green fluorescence signal visualized in transgenic citrus roots. **(C)** Genomic PCR identification of the regenerated hairy roots using *Cas9*-specific primers (upper panel) and *GFP*-specific primers (lower panel). +: plasmid (used as a positive control); -: ddH_2_O; WT: wild-type, 1-8/1-7: transgenic lines of hairy roots. **(D)** Alignment of the gene-edited sequence of transgenic hairy roots with the gRNA-targeted loci. Orange nucleotides indicate gRNAs and blue nucleotides indicate PAMs. The scale bar indicates 1 cm.

In recent years, the CRISPR/Cas9 system has been well developed as a powerful gene editing tool and become the new prominent technology on the gene functional research (Jiang and Doudna, 2017; Manghwar et al., 2019; Rönspies et al., 2022). This technology accelerates crop improvement and biological research in various species, as well as in *Citrus* (Zhu et al., 2019; Debernardi et al., 2020; Su et al., 2023). However, due to the low transformation efficiency and time-consuming features of *Citrus*, using low-efficiency CRISPR/Cas9 system when performing gene editing would enormously retard research progress. Therefore, it is necessary to screen high-editing-efficiency CRISPR/Cas9 system before genetic transformation. Here we used the *Citrus* seeds hairy root system to verify gene editing efficiency and mutations. As a proof-of-concept for CRISPR editing applications in citrus seed hairy root transformation system, an endogenous gene (*CsPP2-1*) was edited. Two gRNA-target sites were created in the first and second exons of the *CsPP2-1*, respectively (**Figure 6A**). A pCAMBIA1300-p*YAO*-Cas9 plasmid harboring Cas9 and an sgRNA expression cassette was used to transform *C. sinensis* and *P. trifoliata*, which manifested as valuable cultivar and rootstock resources, respectively. Thirty days after transformation, transgenic hairy roots were selected to investigate the gene editing efficiency. Two primers designed specifically for Cas9 protein and GFP protein were used to identify the DNA extracted from hairy roots emitting green fluorescence. The confirmed positive DNA were amplified using gene-specifc primers to obtain the target sequence for sequencing. Fluorescence microscopy imaging of the transformed hairy roots showed that transgenic hairy roots successfully expressing GFP protein were obtained in both *C. sinensis* and *P. trifoliata* (**Figure 6B**). The PCR amplification further verified that all the hairy roots emitting green fluorescence contained sequences encoding Cas9 protein and GFP protein (**Figure 6C**), indicating that it is reliable to screen positive transgenic hairy roots through fluorescence observation in *A. Rhizogenes*-mediated hairy root transformation system in *Citrus* seeds.

Sequence analysis revealed that transgenic hairy roots from both *C. sinensis* (marked as Cs-1~8) and *P. trifoliata* (marked as Ptr-1~7) transformed with the *CsPP2-1*-pCAMBIA1300-*pYAO :* Cas9 vector had been successfully edited at different targets with gRNA1 and gRNA2. The missing fragments in edited transgenic roots were mainly located between the PAM sequences of gRNA1 and gRNA2, or at the 5’ end of the PAM sites, which is coincident with the properties of Cas9-mediated gene editing. No editing effect could be detected in Ptr-2, among all transgenic hairy roots analyzed, with an overall editing efficiency of 94.1% (**Figure 6D**). A total of 10 homozygous mutations, namely Cs-1/4/5/6/8 and Ptr-1/4/5/6/7, was identified. Three transgenic hairy roots of Cs-6, Ptr-1 and Ptr-3 exhibited editing effect only at one gRNA-targeted loci (gRNA2), while others displayed editing effect at both gRNA-targeted sites. Notably, most of the transformants showed large block deletion mutations, indicating that knockouts achieved were successful and efficient. Therefore, the *A. Rhizogenes*-mediated hairy root transformation system in *Citrus* seeds is a capable tool in high-throughput screening of gRNA targets in CRISPR/Cas9 gene editing system.

## Discussion

Currently, citrus genetic transformation mainly relies on *A*. *tumefaciens*-mediated transformation in citrus epicotyl, the procedure is time-consuming, laborious, genotype-dependent, and hindered by low transformation frequency, which is suboptimal for functional studies of citrus genes. The traditional transformation mediated by *A. tumefaciens* takes 3-5 months from sowing seeds, culturing transformation materials of epicotyl to finally obtaining transgenic regeneration buds, and it requires months or even longer to culture the regenerated buds to enable gene functional validation (Khan et al., 2012). This process mostly needs to be accomplished under an aseptic condition, with high technical requirements and a high possibility of material contamination. Hence, using *A. rhizogenes* to induce transformed hairy roots presents a novel approach in citrus gene functional researches. Previous studies have shown that hairy roots could be induced from several explant types (e.g., internodal stem segments, branches and epicotyls) of citrus and have been demonstrated to be effective for analyzing gene function in citrus (Pérez-Molphe-Balch and Ochoa-Alejo, 1998; Ma et al., 2022; Ramasamy et al., 2023). However, most of the previous studies used mature branches or seedlings of citrus as the explants for hairy root induction, the high differentiation of which would reduce the transformation efficiency and the preliminary planting of seedlings was time-consuming. In light of the protocol reported previously, we consider that there is still potential for optimization of the citrus hairy root transformation system. Therefore, a faster, simpler and more efficient citrus hairy root transformation system using citrus seeds as a starting materials is described here. In this study, we achieved a high frequency of hairy root transformation in four citrus genotypes by developing the transformation procedure. Subsequently, through the exploration and optimization of transformation parameters, the transformation frequency of *C*. *sinensis*, which carries the lowest transformation frequency of 52.3%, was successfully increased to 71.4%. The entire transformation process takes only 15 days to obtain transgenic hairy roots capable for gene functional analysis, which provides an advantage over traditional transformation for studying the biological functions of citrus genes.

### Citrus seeds hairy root transformation system yields high transformation efficiency in a variety of citrus genotypes

As a tool for molecular biology research, research reports on herbaceous plants such as tomato, maize, soybean, and cotton have been well established in recent years using the hairy root system to study gene function and root biology (Ron et al., 2014; Gautam et al., 2018; Huang et al., 2022; Zhou et al., 2022). However, no detailed report on the functional relevance of *A. rhizogenes*-mediated endogenous genes in most non-model plants, especially citrus, is available. Researchers have to utilize transient expression methods or heterologous expression in model plants to analyze gene function, or time-consuming traditional transformation methods to obtain homologous expressing plants. Either way, accurate verification of the original function of genes or screening of an extensive set of candidate genes is difficult. This has resulted in the research on difficult-to-transform but economically valuable plants lagging behind the model plants. In this study, we developed a novel method for hairy root transformation system in citrus seeds and generalized it to four different citrus genotypes including *C*. *sinensis*, *P*. *trifoliata*, *C*. *limon*, and *C*. *grandis*. Most citrus varieties showed efficient root regeneration, with the highest positive rate of 96.1% for *C. limon* (**Figure 2**, **Table 1**). As the most widely grown and economically valuable species in citrus, *C*. *sinensis* possesses a positive rate of only 52.3%. In order to improve the efficiency of hairy root transformation in *C*. *sinensis*, we mapped out the optimization conditions of the transformation process.

The results revealed that the concentration of *Agrobacterium* during the process had a greater effect on the transformation efficiency of *C*. *sinensis* seeds mediated by *A. rhizogenes* compared to the AS concentration. When the *Agrobacterium* concentration was increased from 0.6 to 1.0, the transformation efficiency decreased dramatically (**Figure 3**). It has been reported that a low concentration of *Agrobacterium* leads to a low population of bacterial colonies, thus affecting the transformation efficiency. While excessive *Agrobacterium* concentration may also reduce the transformation efficiency by decreasing the colony activity due to insufficient nutrient supply (Niazian et al., 2023; Purwantoro et al., 2023). Hence, we determined the optimal conditions for *A. rhizogenes*-mediated transformation in *C*. *sinensis* seeds to be OD_600 =_ 0.6 and AS concentration with100 μM, which increased the positive rate to 71.4%.

In conclusion, the hairy root transformation system developed in this study yielded high transformation efficiencies in a variety of citrus species, providing the possibility of screening and validating a wide range of citrus candidate genes and their functions in a more rapid, efficient and broad-spectrum manner.

### Citrus seeds hairy root transformation system leads to more precise subcellular localization

Currently, subcellular localization analysis of proteins in citrus is usually investigated through *A. tumefaciens*-mediated transient transformation of tobacco leaves. The advantages of this system are simple operation and high protein expression. However, the heterologous expression of genes may not reflect their authentic localization in the native species. Therefore, we investigated the subcellular localization of citrus endogenous proteins in the nucleus, membrane and ER using the hairy root transformation procedure. Transgenic hairy roots exhibited GFP fluorescence and subcellular localization of proteins in the nucleus, plasma membrane and ER. Compared to tobacco system, hairy roots had similar protein localization and the cellular localization could be observed more clearly. Of particular note, proteins that exhibited membrane and nuclear localization in tobacco leaves only showed specific membrane localization in citrus hairy roots (**Figure 4**). Similar situations occurred in the subcellular localization analysis of soybean proteins in hairy roots, mCherry-CAAX localized to the cell membrane in soybean hairy roots but to both the membrane and the nucleus in tobacco leaves (Cheng et al., 2021), suggesting that some organisms exhibit taxon-specific subcellular localization of proteins. According to our results, the hairy root system is a powerful tool for studying the subcellular localization of citrus proteins. This system facilitates the identification of gene functions and also has the potential to investigate citrus protein-DNA and protein-RNA interactions.

### Applicability of citrus seeds hairy root system for genetic overexpression and gene editing

At present, most of the citrus gene overexpression and gene editing technologies are accomplished using *A. tumefaciens*-mediated transformation in citrus epicotyl, which is time-consuming, inefficient and marred by the existence of chimeras (Xu et al., 2013; Dai et al., 2018; Basu et al., 2022). In addition, there is a possibility that new mutants generated by the CRISPR/Cas9 system may select inappropriate gRNA targets, leading to failed editing and thus affecting the progress of citrus gene functional research. Thus, considering the rapidity and efficiency of citrus hairy root transformation, the acquisition of gene overexpressing and gene editing materials with hairy root transformation constitutes a better choice for conducting research on the biological functions of citrus genes. Overexpression of *CsWRKY17* in *C*. *sinensis* seeds obtained abundant hairy roots 30 days after transformation, and the expression levels was increased by 10.24 to 218.19-fold compared with the WT (**Figure 5C**). The overall editing efficiency of the *CsPP2-1* gene editing material obtained using CRISPR/Cas9 technology reached 94.1% (**Figure 6C**). Furthermore, hairy roots induced by *A. rhizogenes* often develop from single cells, which results in a lower incidence of chimerism in transgenic hairy roots (Roychowdhury et al., 2017). Nevertheless, a major limitation of this protocol is that only hairy roots can be transformed, but not other citrus tissues. Therefore, this is more advantageous for investigating the cell biological functions of genes in citrus roots than in shoots. Additionally, using this technology with hairy root transformation is a better option to screen high-efficiency editing of gRNA targets before performing genetic transformation of scion material in citrus.

In summary, we established a rapid, universal, and highly efficient hairy root transformation system mediated by *A. rhizogenes* based on the radicles germinating directly from the seeds for the first time, which can be employed for subcellular localization, gene overexpression and gene editing analysis. We anticipate that this gene transformation technology will open up entirely new areas in the application of citrus endogenous genes.

## Data availability statement

The original contributions presented in the study are included in the article/**Supplementary Material**. Further inquiries can be directed to the corresponding author.

## Author contributions

MW: Conceptualization, Funding acquisition, Investigation, Methodology, Supervision, Validation, Writing – review & editing. Y-YQ: Data curation, Formal Analysis, Investigation, Writing – original draft. N-NW: Data curation, Formal Analysis, Writing – review & editing. H-YX: Data curation, Formal Analysis, Writing – review & editing. W-SD: Conceptualization, Funding acquisition, Investigation, Methodology, Supervision, Validation, Writing – original draft.

## References

[B1] Basu S. Sineva E. Nguyen L. Sikdar N. Park J. W. Sinev M. . (2022). Host-derived chimeric peptides clear the causative bacteria and augment host innate immunity during infection: A case study of HLB in citrus and fire blight in apple. Front. Plant Sci. 13. doi: 10.3389/fpls.2022.929478 PMC9816411 36618616

[B2] Castellanos-Arévalo A. P. EstradaLuna A. A. CabreraPonce J. L. ValenciaLozano E. de HerreraUbaldo H. Folter. S. . (2020). Agrobacterium rhizogenes-mediated transformation of grain (Amaranthus hypochondriacus) and leafy (A. hybridus) amaranths. Plant Cell Rep. 39 (9), 1143–1160. doi: 10.1007/s00299-020-02553-9 32430681

[B3] Cheng Y. Y. Wang X. L. Cao L. Ji J. Liu T. F. Duan K. X. (2021). Highly efficient Agrobacterium rhizogenes-mediated hairy root transformation for gene functional and gene editing analysis in soybean. Plant Methods 17 (1), 73. doi: 10.1186/s13007-021-00778-7 34246291 PMC8272327

[B4] Dahro B. Li C. L. Liu J. H. (2023). Overlapping responses to multiple abiotic stresses in citrus: from mechanism understanding to genetic improvement. Hortic. Adv. 1, 4. doi: 10.1007/s44281-023-00007-2

[B5] Dai W. S. Peng T. Wang M. Liu J. H. (2023). Genome-wide identification and comparative expression profiling of the WRKY transcription factor family in two Citrus species with different Candidatus Liberibacter Asiaticus susceptibility. BMC Plant Biol. 23 (1), 159. doi: 10.1186/s12870-023-04156-4 36959536 PMC10037894

[B6] Dai W. S. Wang M. Gong X. Q. Liu J. H. (2018). The transcription factor FcWRKY40 of Fortunella crassifolia functions positively in salt tolerance through modulation of ion homeostasis and proline biosynthesis by directly regulating SOS2 and P5CS1 homologs. New Phytol. 219 (3), 972–989. doi: 10.1111/nph.15240 29851105

[B7] Debernardi J. M. Tricoli D. M. Ercoli M. F. Hayta S. Ronald P. Palatnik J. F. . (2020). A GRF-GIF chimeric protein improves the regeneration efficiency of transgenic plants. Nat. Biotechnol. 38 (11), 1274–1279. doi: 10.1038/s41587-020-0703-0 33046875 PMC7642171

[B8] Estrada-Navarrete G. Alvarado-Affantranger X. Olivares J. E. Guillén G. Díaz-Camino C. Campos F. . (2007). Fast, efficient and reproducible genetic transformation of Phaseolus spp. by Agrobacterium rhizogenes. Nat. Protoc. 2 (7), 1819–1824. doi: 10.1038/nprot.2007.259 17641650

[B9] Fan Y. L. Zhang X. H. Zhong L. J. Wang X. Y. Jin L. S. Lyu S. H. (2020). One-step generation of composite soybean plants with transgenic roots by Agrobacterium rhizogenes-mediated transformation. BMC Plant Biol. 20 (1), 208. doi: 10.1186/s12870-020-02421-4 32397958 PMC7333419

[B10] Fang H. Shi Y. Liu S. Jin R. Sun J. Grierson D. . (2023). The transcription factor CitZAT5 modifies sugar accumulation and hexose proportion in citrus fruit. Plant Physiol. 192 (3), 1858–1876. doi: 10.1093/plphys/kiad156 36911987 PMC10315291

[B11] Gautam M. Elhiti M. Fomsgaard I. S. (2018). Maize root culture as a model system for studying azoxystrobin biotransformation in plants. Chemosphere 195, 624–631. doi: 10.1016/j.chemosphere.2017.12.121 29287271

[B12] Huang P. Lu M. Li X. Sun H. Cheng Z. Miao Y. . (2022). An efficient Agrobacterium rhizogenes-mediated hairy root transformation method in a soybean root biology study. Int. J. Mol. Sci. 23 (20), 12261. doi: 10.3390/ijms232012261 36293115 PMC9603872

[B13] Irigoyen S. Ramasamy M. Pant S. Niraula P. Bedre R. Gurung M. . (2020). Plant hairy roots enable high throughput identification of antimicrobials against Candidatus Liberibacter spp. Nat. Commun. 11 (1), 5802. doi: 10.1038/s41467-020-19631-x 33199718 PMC7669877

[B14] Jiang F. Doudna J. A. (2017). CRISPR-Cas9 structures and mechanisms. Annu. Rev. Biophys. 46 (1), 505–529. doi: 10.1146/annurev-biophys-062215-010822 28375731

[B15] Kereszt A. Li D. Indrasumunar A. Nguyen C. D. Nontachaiyapoom S. Kinkema M. . (2007). Agrobacterium rhizogenes-mediated transformation of soybean to study root biology. Nat. Protoc. 2 (4), 948–952. doi: 10.1038/nprot.2007.141 17446894

[B16] Khan E. U. Fu X. Z. Liu J. H. (2012). Agrobacterium-mediated genetic transformation and regeneration of transgenic plants using leaf segments as explants in Valencia sweet orange. Plant Cell Tissue Organ Cult. 109 (2), 383–390. doi: 10.1007/s11240-011-0092-7

[B17] Liu X. Gong Q. Zhao C. Wang D. Ye X. Zheng G. . (2022). Genome-wide analysis of cytochrome P450 genes in Citrus clementina and characterization of a CYP gene encoding flavonoid 3’-hydroxylase. Hortic. Res. 10 (2), uhac283. doi: 10.1093/hr/uhac283 36818367 PMC9930397

[B18] Luan Q. L. Zhu Y. X. Ma S. J. Sun Y. H. Li X. Y. Liu M. J. . (2021). Maize metacaspases modulate the defense response mediated by the NLR protein Rp1-D21 likely by affecting its subcellular localization. Plant J. 105 (1), 151–166. doi: 10.1111/tpj.15047 33107667

[B19] Ma H. Meng X. Xu K. Li M. Gmitter F. G. Jr. Liu N. . (2022). Highly efficient hairy root genetic transformation and applications in citrus. Front. Plant Sci. 13. doi: 10.3389/fpls.2022.1039094 PMC9647159 36388468

[B20] Manghwar H. Lindsey K. Zhang X. L. Jin S. X. (2019). CRISPR/Cas System: Recent advances and future prospects for genome editing. Trends Plant Sci. 24 (12), 1102–1125. doi: 10.1016/j.tplants.2019.09.006 31727474

[B21] Niazian M. Belzile F. Curtin S. J. de Ronne. M. Torkamaneh D. (2023). Optimization of in *vitro* and ex vitro Agrobacterium rhizogenes-mediated hairy root transformation of soybean for visual screening of transform-ants using RUBY. Front. Plant Sci. 14. doi: 10.3389/fpls.2023.1207762 PMC10361064 37484469

[B22] Pérez-Molphe-Balch E. Ochoa-Alejo N. (1998). Regeneration of transgenic plants of Mexican lime from Agrobacterium rhizogenes-transformed tissues. Plant Cell Rep. 17 (8), 591–596. doi: 10.1007/s002990050448 30736509

[B23] Purwantoro A. Irsyadi M. B. Sawitri W. D. Fatumi N. C. Fajrina S. N. (2023). Efficient floral dip transformation method using Agrobacterium tumefaciens on Cosmos sulphureus Cav. Saudi. J. Biol. Sci. 30 (7), 103702. doi: 10.1016/j.sjbs.2023.103702 PMC10362457 37485451

[B24] Qu X. Christ B. J. (2007). *In vitro* culture of the obligate parasite Spongospora subterranea (cercozoa; plasmodiophorida) associated with root-inducing transferred-DNA transformed potato hairy roots. J. Eukaryot. Microbiol. 54 (6), 465–467. doi: 10.1111/j.1550-7408.2007.00289.x 18070323

[B25] Ramasamy M. Dominguez M. M. Irigoyen S. Padilla C. S. Mandadi K. K. (2023). Rhizobium rhizogenes-mediated hairy root induction and plant regeneration for bioengineering citrus. Plant Biotechnol. J. 21 (9), 1728–1730. doi: 10.1111/pbi.14096 37314751 PMC10440979

[B26] Ron M. Kajala K. Pauluzzi G. Wang D. Reynoso M. A. Zumstein K. . (2014). Hairy root transformation using Agrobacterium rhizogenes as a tool for exploring cell type-specific gene expression and function using tomato as a model. Plant Physiol. 166 (2), 455–469. doi: 10.1104/pp.114.239392 24868032 PMC4213079

[B27] Rönspies M. Schindele P. Wetzel R. Puchta H. (2022). CRISPR-Cas9-mediated chromosome engineering in Arabidopsis thaliana. Nat. Protoc. 17 (5), 1332–1358. doi: 10.1038/s41596-022-00686-7 35388178

[B28] Roychowdhury D. Halder M. Jha S. (2017). Agrobacterium rhizogenes-mediated transformation in medicinal plants: genetic stability in long-term culture. In S. Jha (Ed.), Transgenesis Secondary Metab. Reference series in phytochemistry. (Cham: Springer). pp. 323–345.

[B29] Song J. Sun P. Kong W. Xie Z. Li C. Liu J. H. (2022). SnRK2.4-mediated phosphorylation of ABF2 regulates ARGININE DECARBOXYLASE expression and putrescine accumulation under drought stress. New Phytol. 238 (1), 216–236. doi: 10.1111/nph.18526 36210523

[B30] Su H. Wang Y. Xu J. Omar A. A. Grosser J. W. Calovic M. . (2023). Generation of the transgene-free canker-resistant Citrus sinensis using Cas12a/crRNA ribonucleoprotein in the T0 generation. Nat. Commun. 14 (1), 3957. doi: 10.1038/s41467-023-39714-9 37402755 PMC10319737

[B31] Wang M. Dai W. S. Du J. Ming R. H. Dahro B. Liu J. H. (2019). ERF109 of trifoliate orange (Poncirus trifoliata (L.) Raf.) contributes to cold tolerance by directly regulating expression of Prx1 involved in antioxidative process. Plant Biotechnol. J. 17 (7), 1316–1332. doi: 10.1111/pbi.13056 30575255 PMC6576027

[B32] Wu G. A. Prochnik S. Jenkins J. Salse J. Hellsten U. Murat F. . (2014). Sequencing of diverse mandarin, pummelo and orange genomes reveals complex history of admixture during citrus domestication. Nat. Biotechnol. 32 (7), 656–662. doi: 10.1038/nbt.2906 24908277 PMC4113729

[B33] Wu G. A. Sugimoto C. Kinjo H. Azama C. Mitsube F. Talon M. . (2021). Diversification of mandarin citrus by hybrid speciation and apomixis. Nat. Commun. 12 (1), 4377. doi: 10.1038/s41467-021-24653-0 34312382 PMC8313541

[B34] Wu G. A. Terol J. Ibanez V. López-García A. Pérez-Román E. Borredá C. . (2018). Genomics of the origin and evolution of Citrus. Nature 554 (7692), 311–316. doi: 10.1038/nature25447 29414943

[B35] Xu Q. Chen L. L. Ruan X. Chen D. Zhu A. Chen C. . (2013). The draft genome of sweet orange (Citrus sinensis). Nat. Genet. 45 (1), 59–66. doi: 10.1038/ng.2472 23179022

[B36] Zhang S. Q. Liang M. Wang N. Xu Q. Deng X. X. Chai L. J. (2018). Reproduction in woody perennial Citrus: an update on nucellar embryony and self-incompatibility. Plant Reprod. 31 (1), 43–57. doi: 10.1007/s00497-018-0327-4 29457194

[B37] Zhang Y. Ming R. Khan M. Wang Y. Dahro B. Xiao W. . (2022). ERF9 of Poncirus trifoliata (L.) Raf. undergoes feedback regulationby ethylene and modulates cold tolerance via regulating a glutathione S-transferase U17 gene. Plant Biotechnol. J. 20 (1), 183–200. doi: 10.1111/pbi.13705 34510677 PMC8710834

[B38] Zhang Y. Zhu J. Khan M. Wang Y. Xiao W. Fang T. . (2023). Transcription factors ABF4 and ABR1 synergistically regulate amylase-mediated starch catabolism in drought tolerance. Plant Physiol. 191 (1), 591–609. doi: 10.1093/plphys/kiac428 36102815 PMC9806598

[B39] Zhou L. Wang Y. Wang P. Wang C. Wang J. Wang X. . (2022). Highly efficient Agrobacterium rhizogenes-mediated hairy root transformation for gene editing analysis in cotton. Front. Plant Sci. 13. doi: 10.3389/fpls.2022.1059404 PMC9832336 36643290

[B40] Zhu C. Zheng X. Huang Y. Ye J. Chen P. Zhang C. . (2019). Genome sequencing and CRISPR/Cas9 gene editing of an early flowering Mini-Citrus (Fortunella hindsii). Plant Biotechnol. J. 17 (11), 2199–2210. doi: 10.1111/pbi.13132 31004551 PMC6790359

[B41] Zou X. Jiang X. Xu L. Lei T. Peng A. He Y. . (2017). Transgenic citrus expressing synthesized cecropin B genes in the phloem exhibits decreased susceptibility to Huanglongbing. Plant Mol. Biol. 93, 341–353. doi: 10.1007/s11103-016-0565-5 27866312

