# Supplementary material for: Highly efficient Agrobacterium rhizogenes-mediated hairy root transformation in citrus seeds and its application in gene functional analysis
Source: Front Plant Sci. 2023 Oct 31;14:1293374. doi: 10.3389/fpls.2023.1293374 (PMC10644275; doi:10.3389/fpls.2023.1293374)
Supplement: Supplementary file 1 [file Table_1.docx]

**Additional file 1: Table S1. List of primer sequences used in this study**

| **Purpose** | **Primer names** | **Primer sequences (5’-3’)** | |
| --- | --- | --- | --- |
|  |  | **Forward primer** | **Reverse primer** |
| Overexpression vector construction | *CsWRKY17*-pCAMBIA1300 | TCTAGAATGGCGGTGGAGCTGATGGGATTTCC | CCCGGGTCAGTGGTGGTGGTGGTGGTGCGTTGACTCGAAAACCAAACC |
| Transgenic identification of overexpression plants | 35S | GACCTAACAGAACTCGCCGT |  |
|  | NPTII | CGGCTATGACTGGGCACAACA | CGGCAGGAGCAAGGTGAGATG |
|  | GFP | GTAAACGGCCACAAGTTC | GGATCTTGAAGTTCACCTT |
| Gene expression analysis | CsActin QRT | CATCCCTCAGCACCTTCC | CCAACCTTAGCACTTCTCC |
|  | *CsWRKY17* QRT | CTCTACGGCGAGTTACGTCC | TCCGGTGATAGCAGAGGACA |
| gRNA design | *CsPP2-1* gRNA1 | ATTGACATCATGAAGCAGAACAGG | AAACCCTGTTCTGCTTCATGATGT |
|  | *CsPP2-1* gRNA2 | ATTGGAGGCAACCATAAAACCCAG | AAACCTGGGTTTTATGGTTGCCTC |
| Transgenic identification of gene editing plants | Cas9 | AGAAGAAGATACACCAGAC | GTTGATGGGGTTTTCCTC |
|  | *CsPP2-1* CRISPR | GCAATTGGGAGAAGCCGACC | GCAAATGTGTAGTCTCCTCCCC |
